# Supplementary material for: Association between oral microbiome and seven types of cancers in East Asian population: a two-sample Mendelian randomization analysis
Source: Front Mol Biosci. 2023 Nov 21;10:1327893. doi: 10.3389/fmolb.2023.1327893 (PMC10702768; doi:10.3389/fmolb.2023.1327893)
Supplement: Supplementary file 1 [file DataSheet1.ZIP › Supplementary Materials/MR plots of saliva/saliva═╝/Gastric cancer/pheno.2239_to_gastric cancer_forest.pdf]

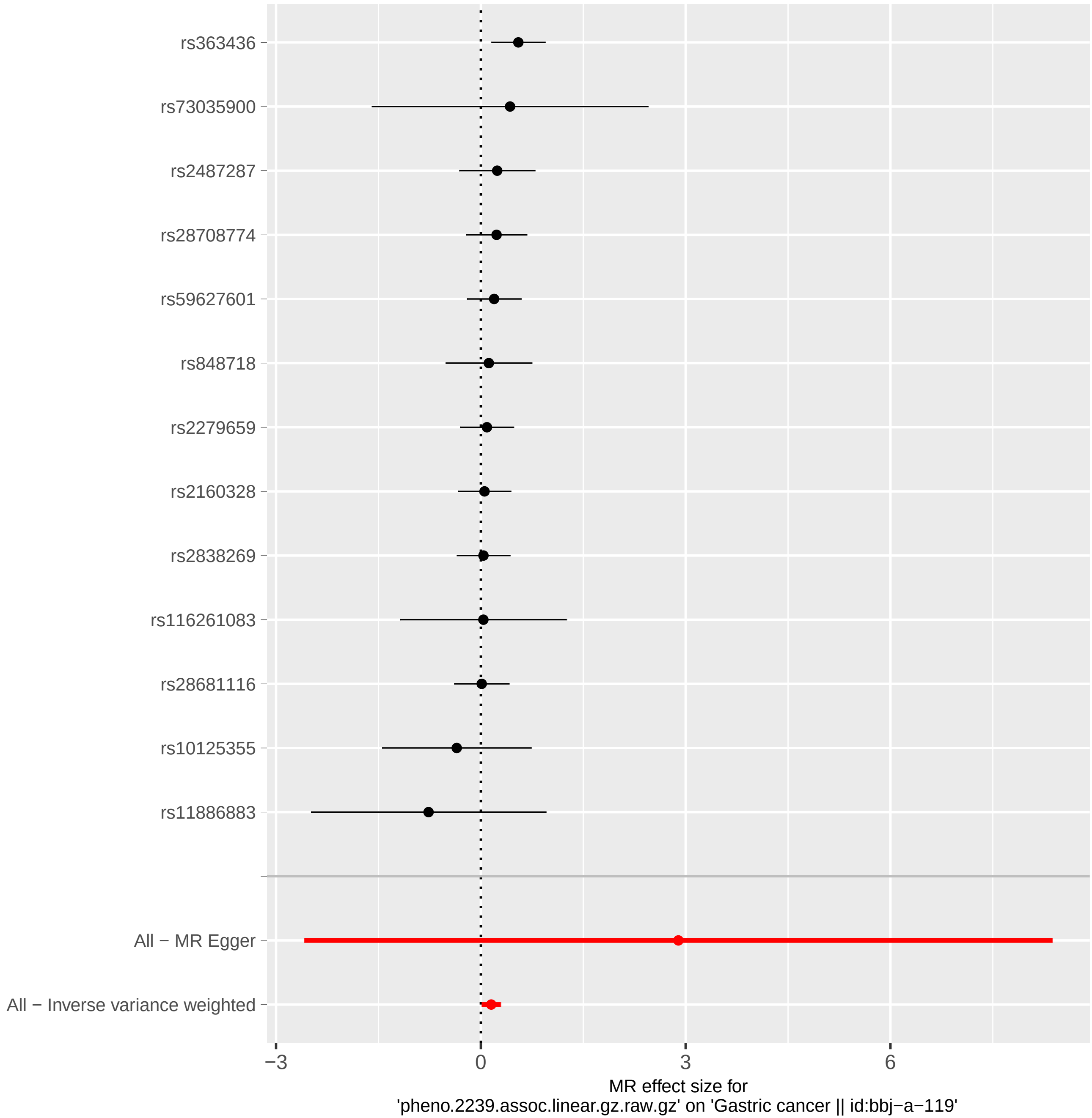

rs363436
rs73035900
rs2487287
rs28708774
rs59627601
rs848718
rs2279659
rs2160328
rs2838269
rs116261083
rs28681116
rs10125355
rs11886883
All − MR Egger
All − Inverse variance weighted
−3
0
3
6
MR effect size for
'pheno.2239.assoc.linear.gz.raw.gz' on 'Gastric cancer || id:bbj−a−119'
